# Implementation of HPV Self‐Sampling in Cervical Cancer Screening Programs: A Bibliometric Analysis of Research Output, Global Trends, and Collaboration Networks

**DOI:** 10.1155/bmri/9345778

**Published:** 2026-05-30

**Authors:** Daysi Diaz-Obregón, Miguel Paco-Fernández, Marysela Ladera-Castañeda, Miguel Huamaní-Contreras, Luis Cervantes-Ganoza, Miriam Castro-Rojas, César Cayo-Rojas

**Affiliations:** ^1^ Research Directorate, Institute for Health Technology Assessment and Research (IETSI), Social Health Insurance of Peru (EsSalud), Lima, Peru; ^2^ School of Stomatology, Universidad Privada San Juan Bautista, Lima, Peru, upsjb.edu.pe; ^3^ Faculty of Medicine, Universidad Nacional Federico Villarreal, Lima, Peru, unfv.edu.pe

**Keywords:** bibliometrics, health services accessibility, mass screening, papillomavirus infections, specimen handling, uterine cervical neoplasms

## Abstract

**Background:**

Cervical cancer is still a significant issue, especially in low‐ and middle‐income countries. Adding HPV self‐sampling to cervical cancer screening programs could make the process simpler for people to receive essential health care and support universal health coverage. Therefore, this study is aimed at mapping research output, global trends, and collaboration networks related to the implementation of HPV self‐sampling in cervical cancer screening programs.

**Methods:**

A bibliometric, descriptive, and retrospective study was performed, covering the period from 2006 to October 2025. A search was conducted in Scopus, confined to the TITLE‐ABS‐KEY fields, without language limitations, and restricted to documents in their final publication stage and original research articles. The search syntax used Boolean operators (AND, OR) to combine four thematic blocks: self‐sampling/self‐collection, etiological agent, cervical screening, and implementation and programmatic delivery. The study examined a number of indicators, such as the volume of research output, the amount of authorship credit per article, the number of normalized citations, the number of citations, the h‐index, the g‐index, and the m‐index. R (RStudio) Version 4.3.1 was used to analyze the data.

**Results:**

There were 824 articles published in 268 journals. The number of research papers published each year went up by 14.90%, and each paper received an average of 21.16 citations per year. Each paper had 8.38 coauthors, and 39.56% of the time, the coauthors were from other countries. The United States and Australia became major contributors, working together in dense networks that crossed continents. Editorial dissemination was primarily found in oncology and public health journals, with cocitation patterns connecting clinical and public health areas. The authorship distribution mirrored Lotka′s pattern, with 71.90% of authors contributing a single article and 4.10% producing five or more. The focus shifted from cytology and acceptability to molecular testing and self‐sampling from 2006 to 2011. From 2012 to 2016, the focus was more on equity and digital support tools along the screening pathway. The focus on sociocultural issues got stronger between 2017 and 2019. From 2020 to 2025, the literature began to emphasize clinical outcomes, HPV‐16, populations living with HIV, and programmatic implementation, including the collection of urine samples.

**Conclusion:**

Research on HPV self‐sampling is expanding, although it remains geographically concentrated. International collaboration is substantial, and the thematic focus has shifted toward programmatic implementation in higher‐risk populations, with increasing emphasis on equity and digital support. These patterns underscore the need for stronger translational partnerships led by stakeholders in high‐burden settings and supported by open science practices to reduce geographic disparities and inform context‐sensitive public policies.

## 1. Background

Cervical cancer remains a significant issue worldwide. In 2022, there were about 660,000 cases and 350,000 deaths from this disease, which occurred mostly in low‐ and middle‐income countries [1]. Persistent infection with high‐risk human papillomavirus (HPV), particularly Genotypes 16 and 18, is the necessary cause of nearly all cervical cancers. Early‐stage cervical cancer may be asymptomatic, whereas more advanced disease can present with abnormal vaginal bleeding, pelvic pain, or foul‐smelling vaginal discharge; depending on stage and clinical context, treatment may include surgery, radiotherapy, chemotherapy, systemic therapy, and palliative care [2, 3]. HPV self‐sampling in cervical cancer screening programs is defined as the collection of vaginal swabs by users themselves for HPV DNA testing as a primary screening method within organized screening schemes, with the potential to reduce barriers to care and expand screening coverage [4, 5]. This strategy is an operational component of the World Health Organization (WHO) Global Initiative for the Elimination of Cervical Cancer and its 90–70–90 targets for 2030 (90% HPV vaccination coverage by age 15, 70% screening coverage at ages 35 and 45 with a high‐performance test, and 90% treatment coverage among women with cervical disease [6]. By expanding access to essential, quality screening services, HPV self‐sampling may also contribute to progress toward universal health coverage (SDG 3.8) [7].

Historically, guidelines have shifted from cytology toward HPV DNA testing as the preferred method, explicitly incorporating HPV self‐sampling as a valid option within organized programs [4, 5]. In the United States, the 2024 draft statement of the US Preventive Services Task Force (USPSTF)—open for public comment—proposes 5‐yearly HPV DNA–based screening every 5 years using samples collected either by the patient or by the clinician in health care settings [8]. In 2024, the US Food and Drug Administration (FDA) expanded the uses of two tests to allow HPV self‐sampling in health care facilities. This was a major step upward for implementation [9]. The FDA approved the Teal Wand in 2025 as the first home‐based device for vaginal self‐collection for screening purposes. This made this option available outside of clinical settings [10, 11].

There is an evident difference in how HPV self‐sampling is used in high‐income countries and nations with fewer resources. Since July 1, 2022, Australia has allowed all participants in the National Cervical Screening Program to do HPV self‐sampling. This is backed by specific operational guidelines and funding mechanisms [12, 13]. The Netherlands has included a self‐sampling kit in its population‐based screening program, which is monitored every year and has user materials in several languages [14, 15]. This is different from countries that have difficulties in operation, regulations, and finances [5, 16, 17].

Even though things have improved significantly, there is still a big difference in equity. The highest rates of incidence and death are found in Southeast Asia, Central America, and sub‐Saharan Africa [5]. In 2022, the age‐standardized incidence in the Americas was 11.5 per 100,000 women (6.4 in North America and 15.1 in Latin America and the Caribbean). This is still a lot higher than the 4 per 100,000 threshold for elimination [17]. The WHO says that in 2022, about 23% of all deaths from cervical cancer happened in the African Region [16]. In addition, women with HIV are about six times more likely to get cervical cancer, which makes them a high‐risk group for screening programs [18]. HPV self‐sampling has shown that it is very acceptable and useful for getting more people to participate, especially those who do not have access to health care, such as Indigenous communities, migrant women, and people who live in remote areas. However, to make it work, it needs to be tailored to the situation and have an effective way to communicate [19].

Programmatic effectiveness has consistently produced favorable results. A meta‐analysis of randomized controlled trials (RCTs) indicated that the direct mailing of HPV self‐sampling kits (“mail‐to‐all”) significantly increases screening participation compared with conventional invitation methods [20, 21]. Furthermore, a pragmatic randomized controlled trial conducted within a US health care system illustrated the efficacy of direct mail for both individuals who were “up‐to‐date” and those who were “overdue” for screening [22]. Nonetheless, deficiencies persist in the connection to diagnosis and treatment, as well as in the operational incorporation of self‐sampling within care pathways, especially in environments with reduced service capacity [23].

Although interest in HPV self‐sampling is increasing, previous bibliometric and scientometric studies have examined broader HPV research, global cervical cancer research, and regional cervical cancer screening trends; however, a focused mapping of the programmatic implementation of HPV self‐sampling through an integrated analysis of research output, collaboration structures, and thematic evolution remains scarce [24–26]. The absence of such analyses limits a strategic view of the field, hindering the identification of regional inequities, the allocation of funding, and the strengthening of international cooperation. In this context, consolidated frameworks and tools such as bibliometrix and VOSviewer are particularly suitable for mapping performance and interactions [27, 28].

Accordingly, this bibliometric study is aimed at mapping research output, global trends, and collaboration networks on the programmatic implementation of HPV self‐sampling in cervical cancer screening programs. It addressed three research questions: (i) How has the global literature evolved over 2006–2025 in terms of volume and impact? (ii) Which actors and collaboration structures shape the field? and (iii) Which thematic trajectories are emerging, and how do they align with implementation needs in high‐burden regions?

## 2. Methods

### 2.1. Study Design

This bibliometric study was conducted and reported in accordance with the Guidance List for Reporting Bibliometric Analyses (GLOBAL) [29]. This descriptive, retrospective bibliometric study examined the scientific literature on HPV self‐sampling/self‐collection for cervical screening and its implementation/programmatic delivery. The search covered the period from 1 January 2006 to the data extraction date (15 October 2025; time zone: America/Lima, Peru). All citation‐based indicators are reported as of 15 October 2025.

### 2.2. Research Objectives and Scope

The primary objective of this study was to map the research output, global trends, and collaboration networks related to the implementation of HPV self‐sampling in cervical cancer screening programs. The bibliometric scope was limited to original research articles indexed in Scopus, published in final form between 2006 and 2025, and focused on self‐sampling/self‐collection for HPV within cervical screening and implementation/programmatic contexts. The analysis emphasized performance indicators, citation impact, collaboration structures, and thematic evolution rather than pooled evaluation of clinical effectiveness outcomes.

### 2.3. Search Strategy

A comprehensive search was performed in Scopus (Elsevier, United States) on 15 October 2025, restricted to the TITLE‐ABS‐KEY fields, with no language restrictions. Scopus was used as the sole data source for this bibliometric analysis. Records were limited to documents in their final publication stage and to original research articles (DOCTYPE = ar). The search syntax combined four thematic blocks using Boolean operators (AND, OR): (i) self‐sampling/self‐collection, (ii) the etiological agent (HPV/VPH/human papillomavirus/hrHPV), (iii) cervical conditions and screening, and (iv) implementation and programmatic delivery. Quotation marks were used for phrases, together with truncation (∗) and spelling variants, to maximize sensitivity while maintaining thematic precision.

The final search strategy was:

TITLE‐ABS‐KEY ((“self‐sampl∗” OR “self sampl∗” OR “self‐sample∗” OR “self sample∗” OR “self‐collect∗” OR “self collect∗” OR “self‐collected” OR “self collected” OR “self‐swab∗” OR “self swab∗” OR autotoma∗ OR “auto‐toma” OR “auto toma” OR “auto‐muestreo” OR “auto muestreo” OR “auto‐hisopado” OR “auto hisopado” OR “auto‐coleta” OR autocoleta OR “autoamostr∗” OR “auto‐prélèvement” OR “auto prélèvement” OR “vaginal self‐sampl∗” OR “auto‐toma vaginal” OR “auto toma vaginal”) AND (HPV OR “human papillomavirus” OR hrHPV OR VPH OR “virus del papiloma humano”) AND (“cervical cancer” OR “cervical neoplas∗” OR “cervical intraepithelial neoplas∗” OR CIN OR “cervical lesion∗” OR (cervic∗ W/3 (cancer OR neoplas∗ OR lesion∗ OR screening)) OR (cervic∗ W/3 (screening OR tamizaje OR cribado OR rastreo OR rastreamento OR dépistage)) OR “cáncer de cuello uterino” OR “cancer de cuello uterino” OR “neoplasias del cuello uterino” OR “câncer do colo do útero” OR “neoplasias do colo do útero” OR “cancer du col de l’utérus” OR “néoplasies du col de l’utérus”) AND (implementation OR programme∗ OR program∗ OR “screening program∗” OR “screening programme∗” OR “programa∗ de tamizaje” OR “programa∗ de cribado” OR “programa∗ de rastreo” OR “programa∗ de rastreamento” OR “programme∗ de dépistage” OR uptake OR coverage OR delivery OR “mail‐out” OR “mail out” OR mailout OR postal OR mailing OR “self‐collection kit” OR “home‐based” OR homebased OR “home based” OR community OR “community‐based” OR “community based” OR “primary care” OR “primary healthcare” OR “primary health care” OR digital OR ehealth OR “e‐health” OR m‐health OR mhealth OR telehealth OR telemed∗)) AND PUBYEAR >2005 AND PUBYEAR <2026 AND DOCTYPE (ar) AND (LIMIT‐TO (PUBSTAGE,"final”)).

This search retrieved 825 records.

### 2.4. Selection Criteria

**Figure 1:**
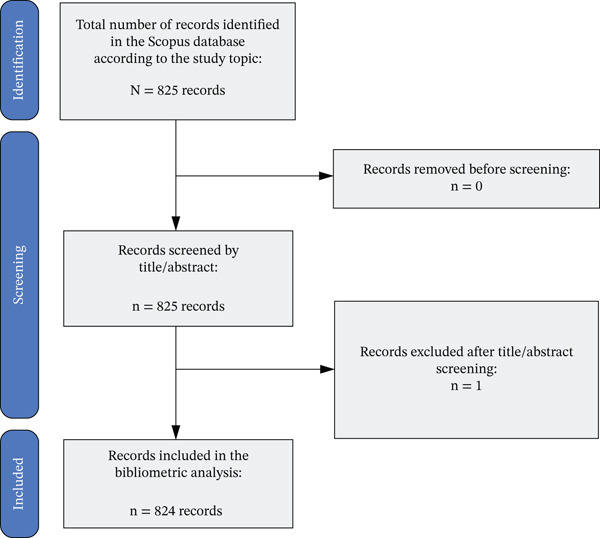
PRISMA‐style flow diagram.

Eligible records were original research articles (DOCTYPE = ar) indexed in Scopus, in the final publication stage, published between 2006 and 2025, with no language restrictions, and addressing HPV self‐sampling/self‐collection in relation to cervical conditions/screening with an implementation or programmatic delivery component. The search was intentionally restricted to original research articles in final publication stage; therefore, other document types, such as review articles, conference papers, editorials, notes, and letters, were not considered. This decision was made to focus the analysis on primary research directly informing programmatic implementation, reduce heterogeneity in document type, and improve comparability across citation‐based and thematic analyses. Titles and abstracts were then thematically screened using predefined eligibility criteria; discrepancies were resolved by consensus between C.C‐R. and L.C‐G. One record (row 805; Chin‐Hong PV et al., Ann Intern Med. 2008; 149(5):300–306. doi:10.7326/0003-4819-149-5-200809020-00004) was excluded because it focused on anal cytology in men who have sex with men rather than cervical screening in women. The final corpus comprised 824 records (Figure 1).

### 2.5. Procedure in Scopus and Bibliometrix/Biblioshiny

Records were exported from Scopus in CSV format as “full records and cited references”, including bibliographic metadata (authors, title, year, source title, volume/issue/pages), citation counts (“Cited by”), identifiers (DOI, Scopus EID), affiliation strings (affiliations; authors with affiliations), author keywords (DE), index keywords (ID), abstract, and references. The export was checked to ensure that all records were classified as Article and in the final publication stage (final). Data were then imported into R 4.3.1 for analysis using the bibliometrix package (via Biblioshiny). Before the trend‐topic and keyword co‐occurrence analyses, the Scopus index keywords field was preprocessed using a predefined stoplist to remove high‐frequency descriptors with low discriminative value for thematic mapping, including document‐type labels, demographic descriptors, study‐design and methodological terms, epidemiological/statistical expressions, clinical‐procedure terms, and broad indexing descriptors (Supporting Information 1). This workflow supported detailed keyword analyses, assessment of publication distributions according to Bradford′s and Lotka′s laws [30, 31], and the generation of visualizations to aid data interpretation.

Because this was a bibliometric study based on bibliographic metadata and science‐mapping procedures, we did not perform standardized full‐text extraction of study‐level operational outcomes, such as HPV assay type, unsatisfactory sample rates, participant literacy or socioeconomic strata, awareness sessions before self‐sampling, turnaround times for results, methods of result communication, HPV positivity rates, or time to colposcopy among HPV‐positive women.

Coauthorship networks (authors/countries; fractional counting) and keyword co‐occurrence networks (DE/ID; full counting) were constructed using a minimum frequency threshold of ≥ 5, together with additional thresholds proportional to corpus size. Matrices were normalized using association strength and clustered using the Louvain community‐detection algorithm.

### 2.6. Data Analysis

Scientific performance, impact, collaboration, and thematic evolution were analyzed. Performance indicators included annual research output and growth rate, as well as productivity by sources, authors, countries, and institutions. Impact indicators included total citations (TC), citations per year per document, and local citation score (LCS) computed within the study corpus; author/country/source impact was further characterized using h‐index, g‐index, and m‐index. Collaboration indicators included the collaboration index, national/international coauthorship, and collaboration networks. Core journals were identified using Bradford′s law, and author productivity patterns were assessed using Lotka′s law. Thematic evolution was modeled using keyword co‐occurrence networks across successive time slices and mapped across periods using the inclusion index described by Cobo et al. interperiod thematic evolution was summarized across four periods aligned with field milestones and balanced by volume/density: P1 (2006–2011), emergence phase (feasibility/acceptability and early validation of self‐sampling); P2 (2012–2016), transition toward HPV primary screening and accumulation of accuracy evidence, including a key regulatory milestone in 2014; P3 (2017–2019), programmatic scale‐up and deployment of postal/home‐based strategies; and P4 (2020–2025), implementation of the 2020 WHO Global Strategy, digital/telehealth acceleration, and further formalization of self‐sampling in guidelines.

### 2.7. Ethical Considerations

This bibliometric study was exempted from protocol review by the Institutional Research Ethics Committee of Universidad Privada San Juan Bautista (letter No. 2636‐2025‐CIEI‐UPSJB), as it was not classified as human subjects research.

## 3. Results

All original research articles on the implementation of HPV self‐sampling in cervical cancer screening programs were published in 268 journals between 2006 and 2025. Annual research output grew by 14.90%. Articles received on average 21.16 citations per year. There was an average of 8.38 coauthors per article, and 7 documents were single‐authored. Overall, 39.56% of all articles involved international coauthorship. The thematic breadth of the field was reflected in 1238 author keywords and 2597 index keywords in Scopus (Table 1).

**Table 1:** Main characteristics of all included articles.

Description	Results
Time period (years)	2006–2025
Sources	268
Documents	824
Annual growth rate (%)	14.90
Average article age (years)	4.96
Average yearly citations per article	21.16
References	4063
Indexed keywords	2597
Author keywords	1238
Authors	4171
Single‐authored documents	7
Coauthors per article	8.38
International coauthorship (%)	39.56

**Figure 2:**
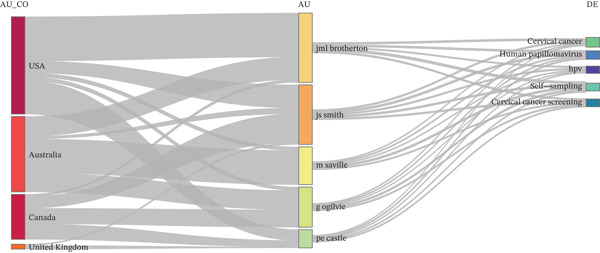
Three‐field plot linking countries, authors, and author keywords, 2006–2025.

The three‐field plot showed a clear geographical concentration of research output in Anglophone countries (United States, Australia, Canada, and the United Kingdom) and a well‐defined core group of authors (Brotherton J.M.L., Smith J.S., Saville M., Ogilvie G., Castle P.E.). The main author keywords converging on these authors (cervical cancer, HPV, self‐sampling, and cervical cancer screening) delineated a stable thematic axis centered on HPV primary screening and HPV self‐sampling as an operational strategy. The thickness of the links suggested a direct country–author–theme relationship, consistent with nationally driven implementation agendas and programmatic evaluations (Figure 2).

**Figure 3:**
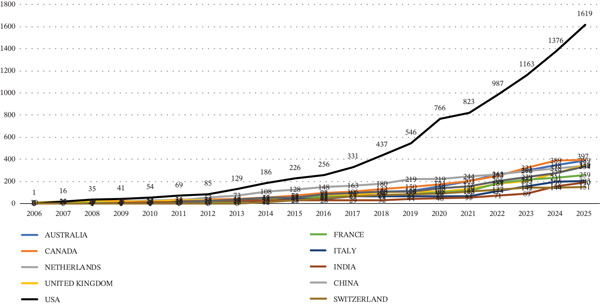
Cumulative country–author occurrences, 2006–2025.

The cumulative country–author occurrence count per year from 2006 to 2025 showed sustained leadership by the United States, with a marked increase from 2016 onward and a clear inflection in output between 2019 and 2020. Australia, the United Kingdom, Canada, the Netherlands, Italy, France, China, and India also displayed upward trajectories, though with a lower slope, as none of these countries exceeded 400 yearly occurrences. In contrast, the United States surpassed 1000 cumulative occurrences from 2023 onwards. Taken together, the pattern suggests a geographic expansion of research on HPV self‐sampling in cervical cancer screening, with a concentration of multinational contributions (Figure 3).

**Figure 4:**
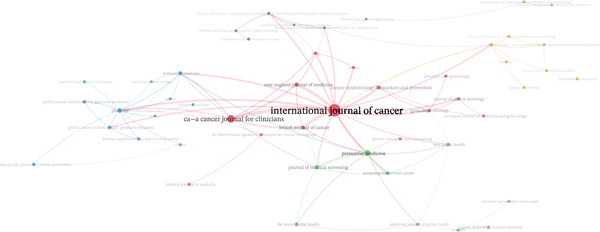
Cocitation network of cited papers.

The Top 10 journals by h‐index placed *International Journal of Cancer* as the leading source (*h* = 19), followed by *BMJ Open* and *PLOS ONE* (both *h* = 15), indicating that these journals host the most consistently cited core of articles on HPV self‐sampling in cervical cancer screening (2006–2025). In terms of volume, *International Journal of Cancer* published the highest number of articles (*N* *P* = 29), followed by *BMJ Open* and *PLOS ONE* (NP = 28 each). Regarding highly cited articles, *International Journal of Cancer* (*g* = 29), *PLOS ONE* (*g* = 26), *BMJ Open* (*g* = 25), and *BMC Public Health* (*g* = 24) stood out. In terms of total citations, *International Journal of Cancer* (TC = 1129) and *Preventive Medicine* (TC = 813) ranked highest. The highest m‐index values were observed for *International Journal of Cancer*, *BMJ Open*, and *BMC Public Health* (*m* = 1.00), reflecting a sustained annual increase in h‐index from the year of their first publications on this topic (Table 2). The cocitation network also showed *International Journal of Cancer* as the most central node, connecting clinical and public health clusters. Additional subgroups were identified around *Preventive Medicine*, *British Journal of Cancer*, and *New England Journal of Medicine*, indicating that the literature integrates both diagnostic evidence and implementation evaluations (Figure 4).

**Table 2:** Volume and citation impact of the Top 10 journals.

Journal	Country	h*-*index	g*-*index	m*-*index	TC	NP	PY‐start
International Journal of Cancer	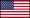	19	29	1.00	1129	29	2007
BMJ Open 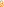	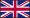	15	25	1.00	652	28	2011
PLOS ONE 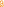	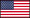	15	26	0.94	698	28	2010
BMC Cancer 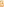	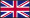	12	17	0.92	455	17	2013
BMC Public Health 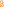	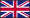	12	24	1.00	579	24	2014
BMC Women′s Health 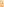	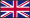	12	18	0.92	333	22	2013
Cancer Epidemiology Biomarkers and Prevention	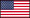	12	16	0.67	510	16	2008
Preventive Medicine	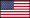	12	16	0.80	813	16	2011
International Journal of Gynecology and Obstetrics	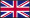	11	19	0.55	466	19	2006
Journal of Medical Screening	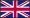	11	18	0.55	537	18	2006

*Note:* 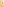 Open access journal.

Abbreviations: g‐index, gives more weight to highly cited articles (detects “hits”). h‐index, number of articles that have at least that number of citations (reflects the “core” of well‐cited articles). m‐index, h‐index adjusted for years since PY‐start, indicating the speed at which a journal accumulates impact on this topic. NP, number of articles on the topic published by the journal. PY‐start, first year in which that journal appears in the dataset for this topic. TC, total citations received by these articles.

**Figure 5:**
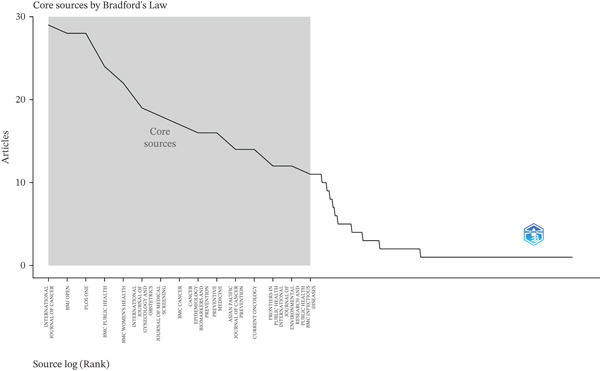
Core journals according to Bradford′s law.

According to Bradford′s law, a compact core of journals (Zone 1) included a small set of titles that accounted for approximately one‐third of all articles, with the five most prominent being *International Journal of Cancer*, *BMJ Open*, *PLOS ONE*, *BMC Public Health*, and *BMC Women′s Health*. Beyond this core, productivity declined sharply, with a long tail of journals publishing only a few articles each. This pattern confirms that dissemination of this topic is channeled through a relatively stable set of oncology and public health journals, which may serve as priority venues for publication and for ongoing surveillance of scientific evidence (Figure 5).

**Figure 6:**
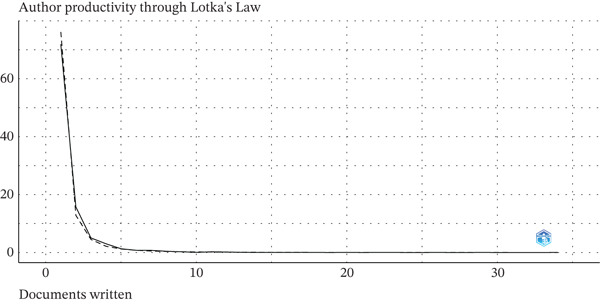
Author productivity according to Lotka′s law.

In line with Lotka′s law, 71.90% of all authors (*n* = 2997) contributed a single article on HPV self‐sampling in cervical cancer screening, whereas 4.10% (186 authors) published five or more articles. The fitted Lotka model (dashed line) overlapped reasonably well with the observed distribution, suggesting a power‐law behavior with an exponent of approximately 2. This indicates that the field relies on a relatively small core of highly productive authors, supported by a wide base of authors contributing occasionally, consistent with a pattern of multi‐institutional and international collaboration (Figure 6).

The Top 10 most productive authors were led by Smith (United States; 34 articles), followed by Brotherton (Australia; 29 articles) and Saville (Australia; 26 articles). Castle (United States; 1508 citations) was the author who was most often cited. The Dutch core also had a lot of citations, with van Kemenade (1246), Meijer (1149), and Berkhof (1135) being the most cited. Saville (*h* = 18) and Castle (*h* = 17) had the most consistent citations. Saville (*g* = 26), Smith (*g* = 25), and Brotherton (*g* = 24) had the most highly cited articles. Saville had the highest m‐index (*m* = 1.39), followed by Castle (*m* = 1.13) and Brotherton (*m* = 1.08). This means that their h‐index has been going up every year since they first published on the subject. Fractional counting (NP/AF) revealed average team sizes of approximately 7–8 authors per article for Saville, Castle, Petignat, and Vassilakos; 8–9 authors for Smith and Berkhof; 9–10 authors for Brotherton and van Kemenade; and 10 or more authors for Ogilvie and Meijer (Table 3).

**Table 3:** Top 10 authors by productivity, citation impact, and collaboration.

Author	Affiliation	Country	h‐index	g‐index	m‐index	TC	NP	PY‐start	AF
Saville M.	Australian Centre for the Prevention of Cervical Cancer	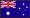	18	26	1.39	723	26	2013	3.30
Castle P.E.	National Cancer Institute (NCI)	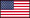	17	22	1.13	1.508	22	2011	2.91
Brotherton J.M.L.	Melbourne School of Population and Global Health	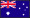	14	24	1.08	602	29	2013	3.17
Smith J.S.	The University of North Carolina at Chapel Hill	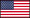	14	25	1.00	672	34	2012	3.91
van Kemenade F.J.	Erasmus MC	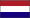	13	16	0.65	1.246	16	2006	1.68
Ogilvie G.	The University of British Columbia	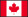	13	23	0.68	563	23	2007	2.26
Meijer C.J.L.M.	Vrije Universiteit Amsterdam	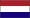	12	12	0.60	1.149	12	2006	1.15
Berkhof J.	Amsterdam UMC ‐ Vrije Universiteit Amsterdam	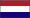	12	14	0.60	1.135	14	2006	1.56
Petignat P.	Université de Genève Faculté de Médecine	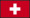	12	19	1.00	398	20	2014	2.41
Vassilakos P.	Geneva Foundation for Medical Education and Research	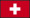	12	19	1.00	398	20	2014	2.41

Abbreviations: AF, fractional authorship credit per article. g‐index, gives more weight to highly cited articles (detects “hits”). h‐index, number of articles that have at least that number of citations (reflects the “core” of well‐cited articles). m‐index, h‐index adjusted for years since PY‐start, indicating the speed at which an author accumulates impact on this topic. NP, number of articles on the topic authored by the given author. PY‐start, first year in which that author appears in the dataset for this topic. TC, total citations received by these articles.

Regarding the Top 10 most influential articles, [21] *The BMJ*) ranked first with 607 total citations, 75.88 citations per year, and a field‐weighted citation impact (FWCI) of 21.73, followed by [32], *Lancet Oncology*) with 560 total citations, 46.67 citations per year, and an FWCI of 13.36. However, the highest annual and field‐weighted impact was observed for [33], *Lancet Oncology*), with 84.50 citations per year and an FWCI of 47.89. Overall, these patterns indicate rapid uptake and dissemination of recent evidence on HPV self‐sampling in cervical cancer screening, grounded in a robust historical base [Table 4].

**Table 4:** Top 10 most influential articles.

Authors (year)	Country	Journal	DOI	TC	TC per year	FWCI
Arbyn et al. (2018)[21]	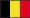	*The BMJ*	10.1136/bmj.k4823	607	75.88	21.73
Arbyn et al. (2014)[32]	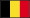	*The Lancet Oncology*	10.1016/S1470‐2045(13)70570‐9	560	46.67	13.36
Bruni et al. (2022)[33]	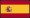	*The Lancet Oncology*	10.1016/S2214‐109X(22)00241‐8	338	84.50	47.89
Gök et al. (2010)[34]	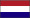	*The BMJ*	10.1136/bmj.c1040	247	15.44	13.19
Lazcano‐Ponce et al. (2011)[35]	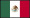	*The Lancet*	10.1016/S0140‐6736(11)61522‐5	194	12.93	14.27
Arrossi et al. (2015)[36]	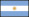	*The Lancet Global Health*	10.1016/S2214‐109X(14)70354‐7	186	16.91	10.66
Serrano et al. (2022)[37]	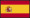	*Preventive Medicine*	10.1016/j.ypmed.2021.106900	183	45.75	18.99
Jerónimo et al. (2014)[38]	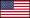	*International Journal of Gynecological Cancer*	10.1097/IGC.0000000000000084	158	13.17	6.43
Herrero et al. (2008)[39]	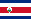	*Vaccine*	10.1016/j.vaccine.2008.07.002	154	8.56	3.44
Bais et al. (2007)[40]	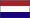	*International Journal of Cancer*	10.1002/ijc.22484	152	8.00	2.27

Abbreviations: FWCI, field‐weighted citation impact; TC, total citations.

**Figure 7:**
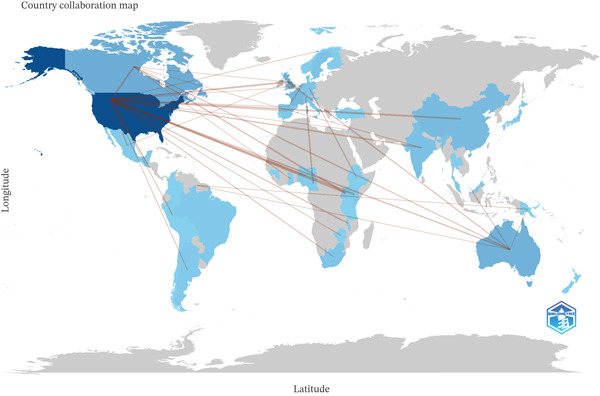
International collaboration map among countries with at least five documents (2006–2025).

A translational collaboration network was evident, with the United States and Australia acting as hubs, concentrating the largest share of scientific production related to HPV self‐sampling in cervical cancer screening. These countries participated in most international links with Western Europe (including the United Kingdom, the Netherlands, Switzerland, among others), as well as relevant connections with the Asia–Pacific region and, to a lesser extent, Latin America and Africa. Among countries with five or more documents on the topic, research output was predominantly generated through transcontinental coauthorship, led by institutions based in high‐income countries, consistent with the international coauthorship rate observed in the corpus (39.56%) (Figure 7).

**Figure 8:**
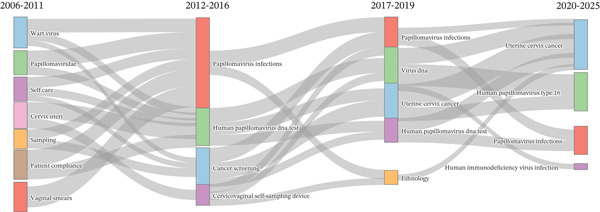
Thematic evolution, 2006–2025.

**Figure 9:**
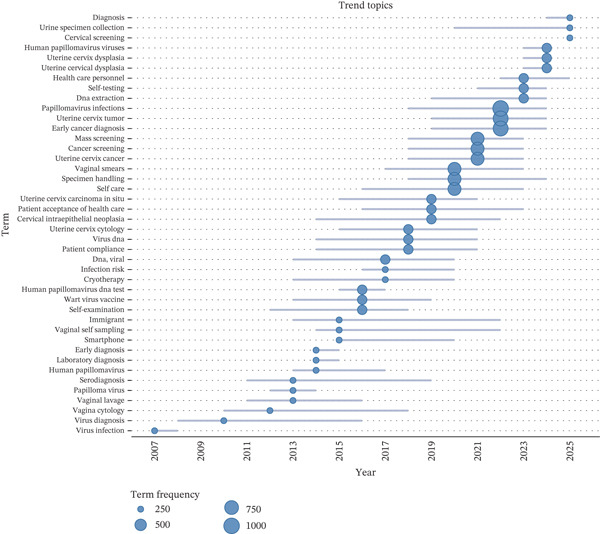
Temporal evolution of topics related to cervical cancer.

Up to 2011, studies focused largely on cytology and acceptability/operational aspects (“vaginal smears”, “sampling”, “patient compliance”, “self‐care”), together with general descriptors of HPV (“wart virus”, “papillomaviridae”). Between 2012 and 2016, the focus shifted toward molecular testing and screening, with an emphasis on “papillomavirus infections”, “HPV DNA test”, and cervicovaginal self‐sampling as a screening method. In this period, the emergence of terms such as “immigrant” and “smartphone” reflected growing attention to equity and digital support within the screening process. Subsequently, between 2017 and 2019, these thematic axes consolidated around “uterine cervix cancer”, and the descriptor “ethnology” emerged, introducing a sociocultural dimension to the acceptance and use of self‐sampling, along with operational signals such as “self‐testing”, “vaginal self‐sampling”, “patient acceptance of health care”, and “specimen handling”. From 2020 to 2025, the focus shifted to clinical outcomes (cervical cancer), etiologic precision (HPV‐16), and higher‐risk populations (HIV infection). There were clear signs of programmatic implementation, like “mass/cervical screening” and “health care personnel,” as well as other ways to do it, like “urine specimen collection.” The research shifted from conventional cytology to molecular detection utilizing self‐sampling, which was progressively incorporated into population‐level cervical screening strategies (Figures 8 and 9).

## 4. Discussion

This bibliometric study provides a comprehensive overview of the incorporation of HPV self‐sampling into cervical cancer screening programs from 2006 to 2025. We analyzed 824 articles and found that research output increased by 14.90% annually. The most impactful citations were in a small group of oncology and public health journals, and the US and Australia led international collaboration networks. The thematic field appears to have rapidly matured and is evidently grounded in a translational agenda. These findings correspond with the programmatic shift from cytology to HPV DNA testing as the primary screening method and with the explicit incorporation of HPV self‐sampling to improve coverage and equity in WHO recommendations [4, 5]. We also discovered regulatory modifications that may facilitate scaling up. For instance, the US FDA approved self‐collection in health care settings in 2024 and gave the first de novo authorization for a home‐based self‐collection device in 2025. This device can be used outside of clinical settings [9, 10]. The evidence shows that HPV self‐sampling works well when done with PCR‐based tests and suggests that more people will participate if proactive outreach strategies are used [20, 21]. Overall, this landscape aligns with the WHO Global Initiative for the Elimination of Cervical Cancer′s 90–70–90 objectives and the aims of Universal Health Coverage. This means that self‐sampling for HPV could be an effective strategy to help underserved groups get the assistance they need [6].

From a perspective of equity and knowledge management, this bibliometric analysis revealed geographic and authorship concentrations that can shape the scientific agenda—showcasing collaboration hubs in the United States and Australia alongside a limited core of highly productive authors—whereas the disproportionate disease burden persists in the African Region, and notable inequities remain in the Americas [17, 41]. This difference, along with the fact that women with HIV are more likely to get cervical cancer, shows how important it is to create HPV self‐sampling models that are sensitive to the situation, along with reliable ways to diagnose and treat the disease [18]. In this context, the increased participation associated with HPV self‐sampling, particularly among nonattenders, suggests the potential to reduce disparities, as improved screening coverage can enhance the likelihood of prompt diagnosis and treatment access in underserved populations. The evidence available supports this view: a community‐based cluster intervention in Argentina increased participation by four times; a community trial in Mexico showed that home‐based self‐sampling was more sensitive than routine cytology; and strategies aimed at nonattenders in the Netherlands effectively increased coverage [34–36].

The patterns that have emerged regarding the process of editorial diffusion suggest a mixed ecosystem in which translational oncology journals coexist with generalist and open‐access outlets. The International Journal of Cancer is a key part of the cocitation network, which aligns with its goal of linking experimental and clinical research [42]. BMJ Open′s editorial practices, such as open peer review and continuous publication, along with PLOS ONE’s methodological standards, which value rigor over novelty, could help make sharing results more open and flexible, along with other similar efforts [43, 44]. When interpreting these patterns, it is advisable to prioritize article content and quality over journal‐based indicators, in line with good practices in research assessment promoted by the DORA Declaration and the Leiden Manifesto [45, 46]. Finally, cocitation networks help visualize communities and knowledge flows and identify nodes that act as bridges; however, they do not substitute for critical appraisal of the validity and applicability of findings [47, 48].

Analysis of the most influential articles in this corpus converged on three complementary axes. First, diagnostic validity and population reach, anchored by meta‐analyses demonstrating the clinical equivalence of self‐sampling versus clinician‐collected samples and reinforced by early evidence of concordance between both sample types in programmatic settings [20, 32, 39]. Second, programmatic adoption and increased coverage, documented by pragmatic studies and trials among nonresponders and in resource‐limited settings (Argentina and Mexico), together with multicenter evaluations and strategies to reach nonattenders [34–36, 40, 38]. Third, policy and monitoring frameworks that catalyze expansion, including comparable estimates of global coverage and syntheses of HPV self‐sampling use in established programs [33, 37]. Overall, these contributions help explain the rapid incorporation of HPV self‐sampling into organized screening, as they provide diagnostic accuracy, demonstrate gains in participation, and offer a comparative scaffold for large‐scale decision‐making [20, 32–37, 39, 40, 38]. Future evidence syntheses should complement bibliometric mapping by systematically examining study‐level implementation outcomes across primary studies, including assay type, sample adequacy, awareness strategies, turnaround times, HPV positivity, and linkage to colposcopy.

To the best of the authors′ knowledge, this study provides the first global bibliometric synthesis focused on HPV self‐sampling in cervical cancer screening, using a reproducible workflow that integrates performance analysis and science mapping via bibliometrix [27]. It further integrates normalization by association strength with Cobo′s thematic evolution approach to identify thematic maturity and trajectories [49–51]. The use of Scopus as a single source favored coverage and standardized metadata; however, coverage and potential indexing biases differ across databases (e.g., Web of Science, Dimensions), and the results should be interpreted in light of these limitations [52–54]. The time periods based on recent normative milestones, such as IARC evaluations and guideline updates that include HPV self‐sampling, help put peaks in research output in the context of policy changes and give program‐relevant information for expanding HPV self‐sampling while keeping quality high [4, 5, 55].

This study has several limitations. Although Scopus was selected as the sole data source for this bibliometric analysis, using only one database may lead to discipline‐, country‐, and language‐related coverage biases, and the results may differ from those obtained from other sources such as PubMed or Web of Science [52–54]. Keyword co‐occurrence reflects semantic proximity but does not supplant other text‐mining methodologies; its efficacy is contingent upon corpus size and preprocessing choices [56]. Citation indicators are also affected by differences in discipline, the age of the article, and delays in time, which means that they need to be carefully interpreted [57, 58]. To mitigate bias, this study refrained from imposing language restrictions and offered a variety of complementary metrics; however, the inherent limitations of single‐source bibliometric designs remain [52–54, 59]. In addition, restricting the corpus to original articles may have excluded emerging findings first disseminated through conference proceedings and high‐level syntheses published as reviews; this decision was made to preserve document‐type comparability in performance and science‐mapping analyses. Future studies could triangulate Scopus with PubMed and Web of Science to assess database‐related variation in coverage and indexing.

In addition, the bibliometric design did not allow pooled evaluation of study‐level implementation indicators, including the most frequently used HPV tests, sample adequacy outcomes, presampling information strategies, turnaround times, result communication pathways, HPV positivity rates, or adherence to colposcopy within program‐defined timeframes.

Based on the findings, it is recommended that the explicit incorporation of HPV self‐sampling into structured screening programs use HPV DNA as the principal assay, emphasizing proactive, population‐based invitation methods, with well‐defined pathways for connection to diagnosis and treatment, and employing PCR‐validated tests [4–6, 20, 21, 34–36]. At the same time, it is important to adhere to the present regulations to make home‐based options and points of care more available. It is also important to make sure that there are strong rules for handling kits and managing results in terms of quality, biosafety, and logistics [34, 42]. To fill in the gaps, interventions should focus on groups that do not get enough help, such as women with HIV, migrant women, and women who live in rural areas. They should use methods that are appropriate for the culture and keep a close eye on coverage and care continuity [17, 18, 36]. We also recommend strengthening pragmatic implementation studies and economic evaluations comparing delivery modalities (home‐based vs. facility‐based), sample types (vaginal vs. urine), and digital components (reminders and follow‐up); in that regard, standardization of indicators and reinforcement of open science and responsible research assessment are needed [45, 46]. Finally, we recommend aligning programmatic monitoring with normative milestones and with the indicators of the 90–70–90 Initiative, incorporating dashboards that link coverage, test quality, turnaround times, and timely treatment. For example, the national integration of HPV self‐sampling into the Dutch cervical cancer screening program since 2017, together with the automatic mailing of kits to women invited at age 30, illustrates this type of programmatic management [5, 6, 14, 15, 33, 60].

## 5. Conclusion

Between 2006 and 2025, the literature on the programmatic implementation of HPV self‐sampling expanded steadily, although research output remained geographically concentrated in a limited number of countries despite substantial international collaboration. A thematic shift was also observed toward implementation in higher‐risk populations, with increasing emphasis on equity and digital support. These patterns highlight the need to strengthen translational and implementation research partnerships with local leadership in high‐burden settings, supported by open science approaches. Overall, strengthening high‐quality collaborative translational research may help inform more robust public policies and facilitate the adaptation of evidence to regional needs.

## Author Contributions

D.D‐O. conceived the research idea. D.D‐O., M.P‐F., M.L‐C., M.H‐C., M.C‐R., L.C‐G., and C.C‐R. drafted the manuscript. C.C‐R. collected the information. M.L‐C., L.C‐G., and C.C‐R. reviewed the study methodology. D.D‐O., M.P‐F., M.L‐C., M.H‐C., M.C‐R., L.C‐G., and C.C‐R. contributed to the interpretation of the findings. All authors critically revised the manuscript.

## Funding

No funding was received for this manuscript.

## Disclosure

All authors read, approved the final version, and agreed to be accountable for all aspects of the work.

## Ethics Statement

This bibliometric study was exempted from protocol review by the Institutional Research Ethics Committee of Universidad Privada San Juan Bautista, under letter No. 2636‐2025‐CIEI‐UPSJB, as the project was not classified as human subjects research.

## Conflicts of Interest

The authors declare no conflicts of interest.

## Supporting information


**Supporting Information** Additional supporting information can be found online in the Supporting Information section. Stoplist used for preprocessing Scopus Index Keywords in the thematic analyses. This file contains the predefined terms removed before the trend‐topic and keyword co‐occurrence analyses, including document‐type labels, demographic descriptors, study‐design and methodological terms, epidemiological/statistical expressions, clinical‐procedure terms, and broad indexing descriptors to improve the specificity and interpretability of the thematic maps.

## Data Availability

The data that support the findings of this study are available from the corresponding author upon reasonable request. The complete search query and the main analytic settings used in biblioshiny are reported in the manuscript, and the stoplist used for the thematic analyses is provided as Supporting Information.
